# Ovariotomy

**Published:** 1853-09

**Authors:** 


OVARIOTOMY.

We learn from Dr. Bradford that, besides the interesting case which is reported in our present number, he has operated successfully in other cases of ovarian disease. Dr. B., we hope, will soon communicate these to the profession. The Committee on Surgery of the Kentucky State Medical Society would be obliged to him for a report of his cases.

